# Response of Five *Miscanthus sinensis* Cultivars to Grasshopper Herbivory: Implications for Monitoring of Invasive Grasses in Protected Areas

**DOI:** 10.3390/plants11010053

**Published:** 2021-12-25

**Authors:** Alina Avanesyan, William O. Lamp

**Affiliations:** Department of Entomology, University of Maryland, 4291 Fieldhouse Drive, 3128 Plant Sciences, College Park, MD 20742, USA; lamp@umd.edu

**Keywords:** grasses, cultivated varieties, invasive plants, plant-insect interactions, plant tolerance, plant resistance, Poaceae, response to herbivores

## Abstract

Introduced grasses can aggressively expand their range and invade native habitats, including protected areas. *Miscanthus sinensis* is an introduced ornamental grass with 100+ cultivars of various invasive potential. Previous studies have demonstrated that the invasive potential of *M. sinensis* cultivars may be linked to seed viability, and some of the physiological traits, such as growth rate. Little is known, however, about whether these traits are associated with response of *M. sinensis* to insect herbivory, and whether plant tolerance and resistance to herbivory vary among its cultivars; which, in turn, can contribute to the invasive potential of some of *M. sinensis* cultivars. To address this issue, in our study we explored the response of five cultivars of *M. sinensis* to herbivory by *Melanoplus* grasshoppers. We demonstrated that plant responses varied among the cultivars during a season; all the cultivars, but “Zebrinus”, demonstrated a significant increase in plant tolerance by the end of the growing season regardless of the amount of sustained leaf damage. Different patterns in plant responses from “solid green” and “striped/spotted” varieties were recorded, with the lowest plant resistance detected for “Autumn Anthem” in the cage experiment. Our results have important applications for monitoring low-risk invaders in protected areas, as well as for biotic resistance of native communities to invasive grasses.

## 1. Introduction

Exotic plant species can pose a substantial threat to protected areas, affecting native and threatened species, altering soil seed bank composition, and representing a further challenge for conservation efforts [1,2,3,4]. Early detection and evaluation of the invasion potential of the exotic plant species is critically important for prioritizing the management and restoration actions, early decision making, and developing the best conservation programs [2,4]. However, our knowledge of the impact of exotic plants in protected areas is often limited to the degree of plant invasion, and the occurrence of invasive plant species in the protected areas, whereas other aspects are still poorly investigated [1,3]. Meanwhile, various plant traits, including but not limited to the plant life form, life strategy, pollination success, and dispersal by birds, are positively associated with exotic species establishment and invasion potential [4,5,6]. This is especially relevant to exotic grasses, which are typically pest and disease-free, and are often used not only for ornamental purposes, but also for water conservation purposes in the protected areas. A recent review by Foxcroft et al. [3] showed that grasses (seven species) comprise 12% of all the highly invasive plants in the protected areas; of these, a substantial part of the species belong to the Poaceae family. The results from our previous work have suggested that it is important to better understand morphological and physiological traits of exotic grasses, and especially their responses to native herbivores which could facilitate the biotic resistance of native communities [7,8].

This study focuses on the Chinese silver grass, *Miscanthus sinensis* Andersson (Poaceae), which is a tall perennial C4 grass, native to East Asia. *Miscanthus sinensis* is a warm-season grass of high forage quality which is adapted to a wide range of climatic conditions including temperate, subtropical, and tropical climates [2,9]. It is found throughout the main islands of Japan in grasslands and upland slopes after burning or mowing [10]. In China, *M. sinensis* is widely distributed and it can also be found in various ecological environments, including river banks and mountains, disturbed habitats, abandoned lands with various soil acidity regimes, as well as volcanic regions [10,11,12,13].

*Miscanthus sinensis* was introduced in the US from Japan in late 1800s for agricultural and horticultural purposes [2], with the first record of planting in North Carolina in 1893–1895 [14,15]. It quickly became a very popular ornamental grass. By 1913, however, *M. sinensis* had escaped cultivation, and by 1940, naturalized populations of *M. sinensis* were recorded in New York, Washington DC, Florida, and West Virginia. By 2014, *M. sinensis* was naturalized in 25 states, especially across the eastern US, producing populations of various size and with wind-dispersed seeds [16]. Between North Carolina and Massachusetts, including Maryland (our study site location), *M. sinensis* colonizes disturbed areas, such as roadsides, open fields, and forest understories, showing high tolerance to various soil and nutrients conditions, as well as a wide range of temperature and humidity [16,17].

*Miscanthus sinensis* is considered to be an invasive grass in a number of states, and a noxious plant in other states as it forms dominant stands [14]. In our previous work, we showed that *M. sinensis* possesses lower plant resistance (measured as leaf damage) and higher or similar plant tolerance (measured as plant growth during both herbivory and a subsequent regrowth period) to grasshopper herbivory compared to native grasses. In our previous work, however, we used a wild type of a non-cultivated variety of *M. sinensis* [7,18,19]. To date, more than 100 varieties of *M. sinensis* were cultivated for ornamental purposes: these cultivars have a wide range of phenotypic variation and adaptability to different environmental conditions [16]. *M. sinensis* cultivars differ in color, size, trichome density, leaf width, and plant architecture, as well as in ability for successful establishment: some of the cultivars produce seeds with no to little viability [20,21]. In addition, *M. sinensis* cultivars might potentially perform various herbivory responses, which in turn might contribute to their invasive potential. To date, however, the invasion potential, and specifically, the response of *M. sinensis* cultivars to insect herbivory, are not well studied, but are very important for bioconservation purposes, and for risk assessment in the protected areas should a certain *M. sinensis* cultivar escapes cultivation.

To address these issues and knowledge gaps, and to continue exploring the invasive potential of *M. sinensis*, we focused on the following main research question: Do *M. sinensis* cultivars of various appearance (e.g., dark/bright coloration, solid/striped patterns, etc.), demonstrate similar tolerance and resistance to common native insect herbivores, such as acridid grasshoppers? Specifically, we were interested in (a) how *M. sinensis* cultivars sustain herbivory from native insect species, and (b) implications of the differences in plant responses (if any) for biotic resistance of native communities to low-impact invaders in protected areas. Based on our previous studies on feeding preferences of insect herbivores [19], we expected these five cultivars to show variation of resistance and tolerance to grasshopper herbivory; we also hypothesized that the cultivars with solid coloration might be less attractive for acridid grasshoppers, and therefore could potentially possess a higher level of invasiveness.

## 2. Results

### 2.1. M. sinensis Tolerance and Resistance to Grasshopper Herbivory: Field Experiment

**Figure 1:**
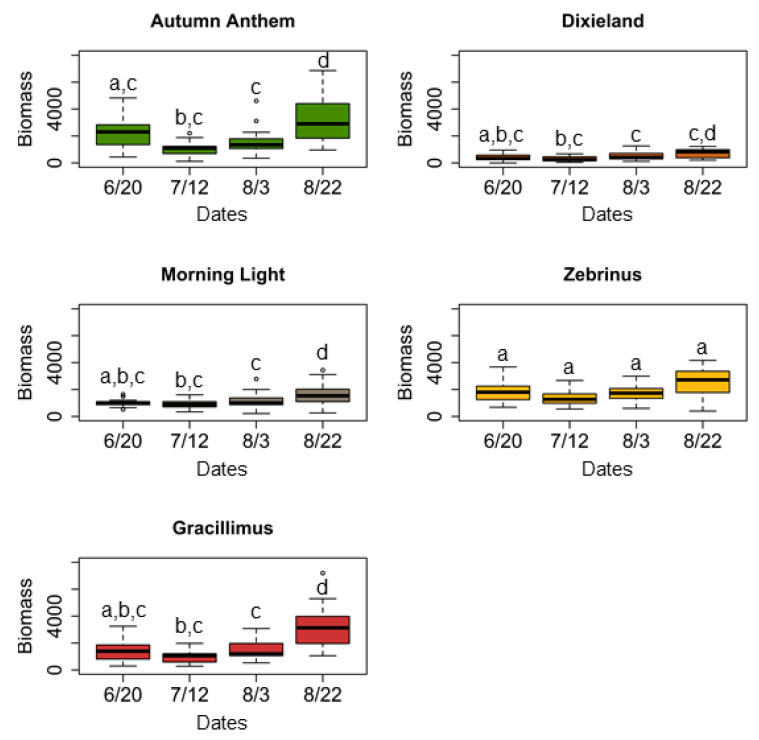
Comparisons of plant biomass changes of *M. sinensis* cultivars during herbivory assays in the field at the Western Maryland Research and Education Center (Keedysville, MD, USA), across four time points during the growing season in 2018. Box plots represent measurements of the product [height × number of leaves], as a proxy for plant biomass, which was used for comparisons. Data comparisons were conducted using one-way repeated measures analysis of variance (ANOVA); plant biomass changes that do not share a letter is significantly different based on post hoc Tukey’s honestly significant difference (HSD) test (Tukey HSD: *p* < 0.05).

In the field experiment, we found that overall plant biomass of *M. sinensis* cultivars varied significantly between the time points (Table 1, Figure 1). “Autumn Anthem” cultivar showed a significant decrease in plant biomass by the second time point in the middle of the season, and then a significant increase in plant biomass by the final time point (i.e., the end of the season); interestingly, the final biomass was significantly higher than the initial plant biomass in the beginning of the season (TukeyHSD: *p* < 0.0001). “Dixieland” cultivar showed a similar pattern with the final plant biomass being significantly higher than that at the beginning of the season and at the second time point (TukeyHSD: *p* < 0.0001); all other pairwise comparisons yielded insignificant results. “Morning light” and “Gracillimus” cultivar showed a significant increase in plant biomass only by the end of the experiment: the final plant biomass was significantly higher than that at all three early time points (TukeyHSD: *p* < 0.0001). “Zebrinus” cultivar, however, demonstrated no significant changes in plant biomass during the season (TukeyHSD: *p* > 0.05).

Since we used a product [height × number of leaves] as a proxy for plant biomass, we were interested in investigating it further and explore whether the height or the number of leaves contributed most to the plant biomass changes. We found that both height and number of leaves of *M. sinensis* cultivars varied significantly between the time points (Table 1). No significant changes in height were recorded for “Dixieland”, and “Morning light” cultivars; “Gracillimus” cultivar showed a significant increase in height by the third time point (in the middle of the season), and then another significant increase by the end of the season (TukeyHSD: *p* < 0.0001); “Autumn Anthem” cultivar demonstrated a slight increase in height by the end of the experiment only. Interestingly, “Zebrinus” cultivar showed a significant difference in height between the initial time point and the third time point only (TukeyHSD: *p* < 0.0001).

We recorded significant changes in the number of leaves, however, for all the cultivars except “Zebrinus” cultivar (Table 1). “Autumn Anthem” demonstrated a significant decrease in the number of leaves by the second time point already which remained the same during the next three weeks in the middle of the season, and then it showed another significant increase in the number of the leaves by the final time point (TukeyHSD: *p* < 0.0001). “Dixieland” and “Gracillimus” cultivars showed repeated significant increases in the number of leaves starting the second time point and up to the end of the season (TukeyHSD: *p* < 0.0001). “Morning light” cultivar, however, demonstrated a significant increase in the number of leaves by the end of the season only (TukeyHSD: *p* < 0.0001).

**Figure 2:**
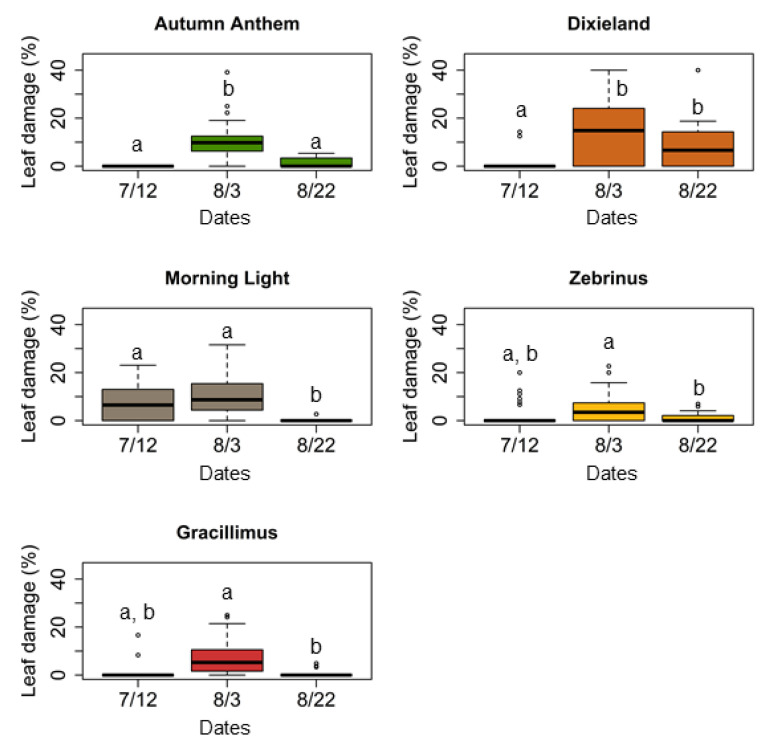
Comparisons of leaf damage in *M. sinensis* cultivars from grasshopper herbivory during herbivory assays in the field at the Western Maryland Research and Education Center (Keedysville, MD, USA), across three time points during the growing season in 2018. Box plots represent measurements of the percentage of apparent leaf damage; leaf damage measurements that do not share a letter is significantly different (Dunn test: *p* < 0.05; the Kruskal–Wallis test followed by post hoc Dunn test).

We also found significant changes in leaf damage of *M. sinensis* cultivars between the time points (Table 1, Figure 2). “Autumn Anthem” showed a significant increase in leaf damage in the middle of the season, and then a significant decrease by the end of the season (Dunn test: *p* < 0.001). “Dixieland” also showed a significant increase in leaf damage in the middle of the season (Dunn test: *p* < 0.001), which then remained the same by the end of the season: no differences in leaf damage were detected between the middle and final time points (Dunn test: *p* > 0.05). “Morning light”, “Gracillimus”, and “Zebrinus” cultivars, however, demonstrated a significant decrease in leaf damage by the end of the season only (Dunn test: *p* < 0.001).

### 2.2. M. sinensis Tolerance and Resistance to Grasshopper Herbivory: Cage Experiment

**Figure 3:**
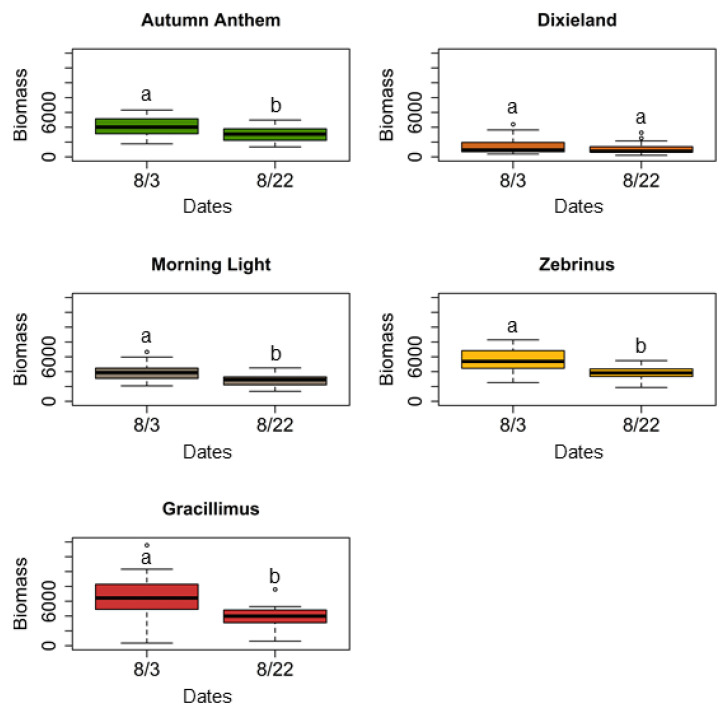
Comparisons of plant biomass changes of *M. sinensis* cultivars during the cage experiment at the Research Plant Growth Facility of the University of Maryland (College Park, MD, USA) in 2018. Box plots represent measurements of the product [height × number of leaves], as a proxy for plant biomass, which was used for comparisons. Data comparisons were conducted using one-way repeated measures ANOVA; plant biomass changes that do not share a letter is significantly different based on post hoc Tukey HSD test (Tukey HSD: *p* < 0.05).

In the greenhouse experiment, all of *M. sinensis* cultivars, except “Dixieland”, showed a significant decrease in plant biomass by the end of the experiment (Table 1, Figure 3). No significant differences were detected in height for either cultivar (Table 1). Comparisons of the number of leaves revealed that only three cultivars demonstrated a significant decrease in this characteristic: “Morning light”, “Gracillimus”, and “Zebrinus” (Table 1).

**Figure 4:**
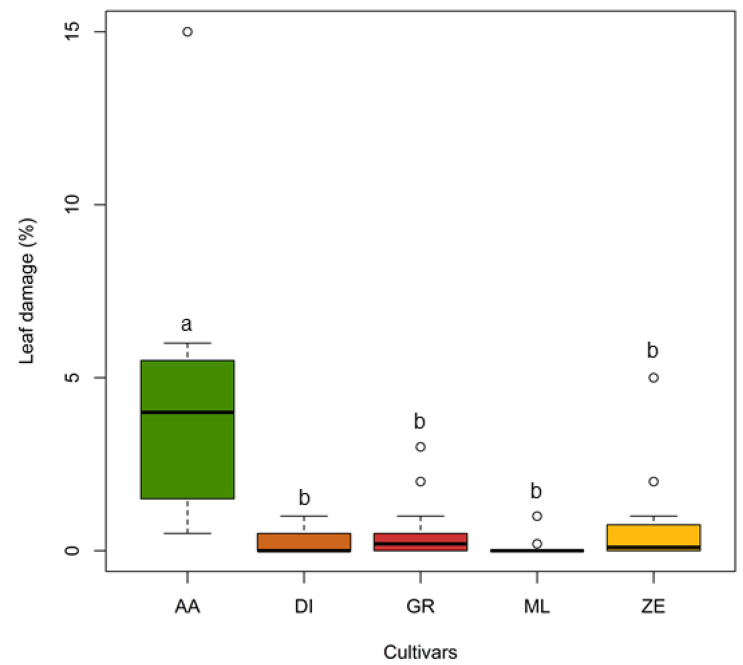
Comparisons of leaf damage in *M. sinensis* cultivars from grasshopper herbivory during the cage experiment at the Research Plant Growth Facility of the University of Maryland (College Park, MD, USA) in 2018. Box plots represent measurements of the percentage of apparent leaf damage in different *M. sinensis* cultivars; leaf damage measurements that do not share a letter is significantly different (Dunn test: *p* < 0.05; the Kruskal–Wallis test followed by post hoc Dunn test was used).

We also found significant differences in leaf damage among *M. sinensis* (Table 1, Figure 4). “Autumn Anthem” showed the highest leaf damage compared to that in other cultivars (Dunn test: *p* < 0.001, Figure 4). Pairwise comparisons between other cultivars yielded insignificant results (Dunn test: *p* > 0.05).

## 3. Discussion

Overall, the results from our study demonstrated that grasshopper herbivory did not suppress the growth of all the *M. sinensis* cultivars used in this study: all the *M. sinensis* cultivars tolerated the grasshopper herbivory well, with either no changes in plant growth (“Zebrinus” cultivar) or substantial increase in plant growth by the end of the season regardless of the amount of leaf damage sustained during the season (“Gracillimus”, “Morning Light”, “Autumn Anthem”, and “Dixieland” cultivars). For the cultivars that demonstrated significant changes in plant growth, we have detected two interesting patterns: (1) solid green varieties, “Gracillimus” and “Morning Light”, showed almost no changes in plant growth during the most of the season, and then a substantial increase in plant biomass at the end of the season (due to both, height increase and increase in the number of leaves); and (2) varieties with striped/spotted coloration, “Autumn Anthem”, and “Dixieland”, showed initial decrease in plant biomass by the middle of the season, which corresponded to the increased leaf damage, and then significant increase in plant biomass by the end of the season due to increase in the number of leaves. A closer look at the targeted herbivory in the experimental enclosure, also showed that “Autumn Anthem” sustained the highest level of leaf damage.

Considering that low resistance and tolerance to insect herbivory might indicate low invasion potential our results suggest a very low invasion risk of “Zebrinus” cultivar at this time. These results support the findings by Meyer and Tchida [20]: the authors explored seed viability of several *M. sinensis* cultivars and showed that “Zebrinus” has the lowest risk (among other cultivars the authors tested) to escape and be invasive in northern climates. However, our findings regarding “Morning Light” are different from that in Meyer and Tchida [20]: based on very high tolerance of this cultivar to grasshopper herbivory exhibited under natural field conditions, our results suggest a high potential of “Morning Light”, as well as “Gracillimus”, for extensive growth and dispersal.

At least three factors could explain high resistance and tolerance of ‘Morning Light’ and “Gracillimus” cultivars to grasshopper herbivory: (a) a joint plant resistance/tolerance response to insect herbivory in general; (b) low visual attractiveness to insect herbivores; and (c) molecular mechanisms which maximize expression of “growth genes”. Regarding plant responses, previous studies proposed that plant resistance and tolerance to insect herbivory could be expressed in a combination [22], and it is possible that plant regrowth after damage (any damage in addition to herbivory) represents “a generalized plant response” [22,23]. Croy et al. [24] in their experiments similarly demonstrated that highly invasive *Phragmites australis* in North America can perform fast growth rates while sustaining herbivory. According to the Evolution of Increased Competitive Ability hypothesis an increased plant growth after damage can be explained by resource allocation from defense to growth [25]. Plant appearance could also be indirectly associated with plant responses to herbivory. Some of the plant morphological and anatomical traits can attract insect herbivores, but some of them (such as trichomes, wax, spines, cell wall thickness, etc.) can serve as a physical barrier and deter insects from feeding [26]. From the insect perspective, the olfactory cues could be very important for insect herbivores during host plant selection [27]. For grasshopper species, in particular, visual cues are highly important for foraging, and choosing a food plant based on plant appearance (especially its striped pattern) is the first step in selection of their food plants [18,19,28]. From the plant perspective, leaf coloration can be associated with plant defence levels to insect herbivory and can, in turn, guide insects to avoid or accept a host plant; specifically, intraspecific variation in leaf color could correlate with insect herbivory levels [27]. Even though leaf variegation could be linked to reduced nutritional value of a plant, our results support the findings by Baisden et al. [29] of the increased feeding of insect herbivores on some of the variegated plant cultivars.

Yet another mechanism which could explain an increased regrowth of “Gracillimus” and “Morning Light” cultivars is a molecular mechanism, such as endoreduplication (i.e., the replication of the genome in the absence of mitosis), which can facilitate the expression of genes responsible for regrowth in response to herbivory [30,31].

Our results also emphasize the importance of native insect herbivores to facilitate the resistance of native communities to exotic plant invasions. In our study all the cultivars sustained leaf damage from grasshoppers, both in the field and in the enclosure, further supporting that native insect herbivores could provide a high pressure on exotic plant species (i.e., biotic resistance hypothesis; [32,33]). According to the biotic resistance hypothesis, native insect herbivores can reduce the success of exotic plant invasions by preferential consumption of exotic plants [33,34]. This information could be valuable for the biological control of invasive grasses in the protected areas, and particularly for additional monitoring of native insect density which was shown to have an impact on biotic resistance mechanisms of native communities [35].

Introduced warm-season C4 grasses can aggressively expand their range and invade native habitats, including protected areas. *Miscanthus sinensis*, as a warm season grass, has a long growing season producing flowers from August to October [20]. While successful self-seeding varies among the cultivars, it is not the only trait that can facilitate *M. sinensis* expansion. The findings from our study suggested that response of *M. sinensis* cultivars to insect herbivory could promote their invasiveness and consequent escape from nursery populations, and require close monitoring especially in close proximity to the protected areas. Specifically, our results suggest the need for monitoring at least two cultivars, “Morning Light” and “Gracillimus”. An additional reason for setting up and developing effective monitoring programs is a high adaptability of introduced and potentially invasive grasses to fire and their high potential to disrupt the grass-fire cycles [36,37]. Finally, close attention to human activities, such as mowing, is needed especially along roadways which is often major pathways for invasive grasses dispersal [38], and where a majority of *M. sinensis* is found [16,20].

## 4. Materials and Methods

### 4.1. Study Sites and Study Species

**Figure 5:**
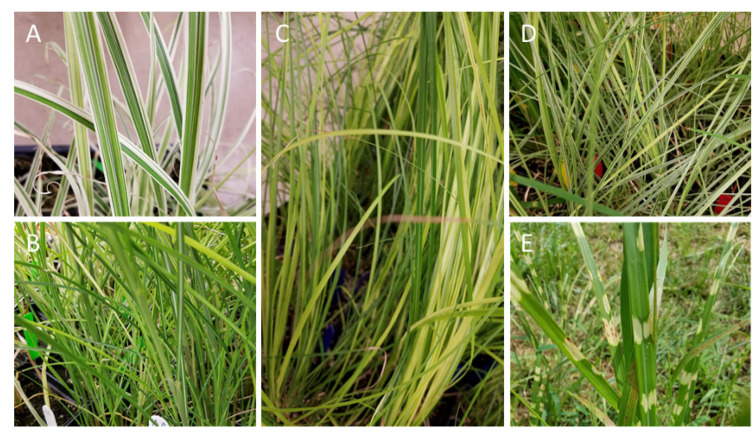
*Miscanthus sinensis* cultivars used in the study: (**A**) “Dixieland”: linear leaves with tapered tips, serrate margins, and white/silver midribs; (**B**) “Autumn Anthem”: grassy green leaves with pointy white spines; (**C**) “Gracillimus”: narrow dark green leaves with white midribs; (**D**) “Morning Light”: bright green leaves with creamy white margins, and (**E**) “Zebrinus”: green leaves with horizontal soft yellow rings.

To explore plant tolerance and plant resistance of *M. sinensis* cultivars to herbivory by native insects, we conducted a one-year study, which included a field experiment and a cage experiment. For these experiments, we used five cultivars of *Miscanthus sinensis* plants, as well as 3rd, 4th, and 5th instars (to accommodate *M. sinensis* growing season) of native *Melanoplus* grasshoppers (Orthoptera: Acrididae). *Melanoplus* grasshoppers were common at our study site and were good candidates to test *M. sinensis* responses to native herbivore insects under natural field conditions. *Melanoplus* grasshoppers were also chosen based on our previous successful herbivore assays with *M. sinensis* in enclosures [7,18,19]. For both the field and cage experiments, we used five cultivars of *M. sinensis*: “Dixieland”, “Autumn Anthem”, “Gracillimus”, “Morning Light”, and “Zebrinus”. These are medium-size *M. sinensis* cultivars which are commonly used for ornamental purposes due to its colorful foliage; they are easy to grow, and they are adaptive to various landscapes and soil. These *M. sinensis* cultivars were also chosen to represent a wide range of coloration, leaf width and texture, and coloration patterns which could potentially “trigger” (attack or repel) feeding preferences of *Melanoplus* grasshoppers: from narrow dark green leaves of *M. sinensis* ‘Gracillimus’, serrate margins and white/silver midribs of *M. sinensis* ‘Dixieland’, to horizontal yellow rings in *M. sinensis* ‘Zebrinus’ (Figure 5A–D).

**Figure 6:**
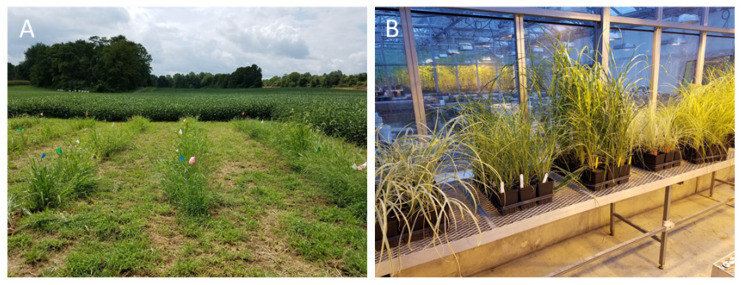
*Miscanthus sinensis* cultivars at the field site, two weeks after planting, and in the greenhouse. (**A**) the field site, *M. sinensis* cultivars during herbivory assays at the Western Maryland Research and Education Center (Keedysville, MD, USA); (**B**) *M. sinensis* cultivars I the greenhouse at the Research Plant Growth Facility of the University of Maryland (College Park, MD, USA) before transferring to the enclosure, for the cage experiment with grasshopper herbivory.

In May 2018, potted plants of five *M. sinensis* cultivars (3.5” grower plugs) were obtained from Santa Rosa Gardens, Gulf Breeze, FL (*M. sinensis* ‘Zebrinus’) and Harris Seeds, Rochester, NY, USA (other four cultivars). After receiving plants from plant nurseries, they were transferred to 4″-pots and allowed to grow for about 2 weeks in the greenhouse at the Research Plant Growth Facility of the University of Maryland (a total of 300 pots; hereafter individual plants). In June 2018, 150 individual plants were transferred to the field site at the Western Maryland Research and Education Center (WMREC, Keedysville, MD, USA) for the field experiment which was conducted during summer 2018 (Figure 6A). The rest of the plants (150 individual plants) were allowed to grow in the greenhouse for another 6 weeks (due to simultaneous presence of 3rd–5th instars of *Melanoplus* grasshoppers later in the season), until the beginning of the cage experiment (Figure 6B). *Melanoplus* grasshoppers, 3rd–5th nymphal instars of *M. femurrubrum* and *M. differentialis*, were then collected at the WMREC and were used in the cage experiment. *Melanoplus* grasshoppers were chosen as native insect herbivores which are (a) widely distributed in the United States, and (b) most commonly occur in the eastern US which contain many protected areas in the states where *M. sinensis* was reported as invasive. In particular, *M. sinensis* was reported invasive in the U.S. National Parks in North Carolina, Virginia, Tennessee, and Washington, DC, USA.

### 4.2. M. sinensis Tolerance and Resistance to Grasshopper Herbivory: Field Experiment

To assess variation (if any) in resistance and tolerance among *M. sinensis* cultivars to grasshopper herbivory under natural conditions in the field, in June 2018 at WMREC we established a plot (16 × 13 m^2^). *Miscanthus sinensis* cultivars (30 individual plants per each cultivar; 150 plants total) were transported from the greenhouse and were randomly planted in six rows at the established field plot; 1.8 m of clear space was allowed between rows, and 1.8 m margins were established around the plot. The grass at the plot margins, as well as between rows, was mowed every two weeks. Each row contained 25 individual plants (five individual plants per cultivar) (Figure 6A). At four different time points during the season, for each individual plant we took the following measurements: (1) plant height (cm); (2) number of leaves; (3) plant biomass; and (4) leaf damage. Following our previous protocols [7], plant biomass was assessed using the product [height × number of leaves] which as we showed previously can be used as a proxy for plant biomass for grasses, and particularly *M. sinensis* (the product [height × number of leaves] explained more than 90% of variation in plant biomass in *M. sinensis* plants).

Only grazed portions left by grasshoppers (i.e., noticeable consumed portions along the midrib) were used to assess the leaf damage. Visual assessment of leaf damage was conducted in both, the field and cage experiments, and the percentage of apparent leaf damage was measured following previous studies [39,40,41]. Multiple categories of leaf damage were established based on leaf damage score following Lieurance and Cipollini [40] but with increments of 10% for damage more than 10%: <1%, 1–2%, 2–5%, 6–10%, 11–20%, 21–30%, 31–40%, 41–50%, etc. with the last category of 91–100%. However, leaf damage more than 50% was not detected for any of the *M. sinensis* cultivars.

### 4.3. M. sinensis Tolerance and Resistance to Grasshopper Herbivory: Cage Experiment

**Figure 7:**
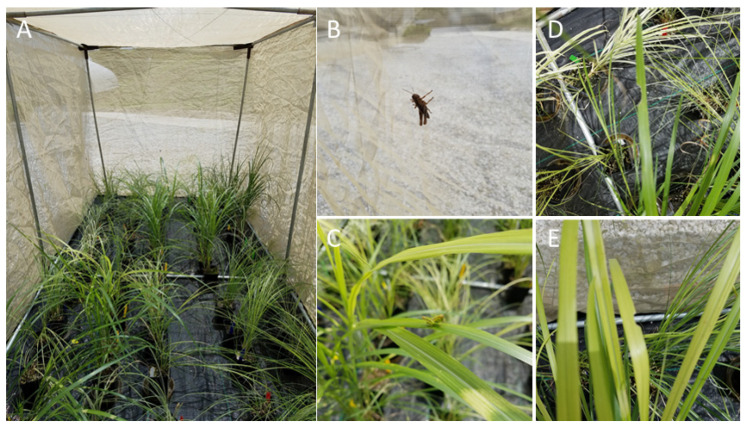
*Miscanthus sinensis* cultivars during the cage experiment at the Research Plant Growth Facility of the University of Maryland (College Park, MD, USA). (**A**) The plants inside the enclosure, 1st day pf the grasshopper herbivory assay; (**B**) A nymph of *Melanoplus* spp. grasshopper on a side wall of the enclosure; (**C**) A nymph of *Melanoplus* spp. grasshopper on “Zebrinus” cultivar inside the enclosure; (**D**,**E**) grazed portion left by grasshopper along the mid-veins of *M. sinensis* cultivars.

About 2 weeks after starting the field experiment, we established one large enclosure (8 × 2 × 3 m^3^) outside the greenhouse complex at the Research Plant Growth Facility of the University of Maryland, and we conducted a choice herbivore assay similar to that in the field with the rest of the potted plants (Figure 7A–E). Similar to the experimental design in the field, we randomly placed 150 individual (potted) plants in 6 rows inside the enclosure (30 pots per plant cultivar), leaving approximately 0.4 m between rows. *Melanoplus* grasshoppers were collected at the WMREC a day before the cage experiment. Following our previous experiments [18,19,42], they were starved for 24 h in the lab, and then 48 nymphs were placed in the enclosure with *M. sinensis* cultivars and were allowed to feed for 3 days. The number of grasshoppers was chosen based on our preliminary grasshopper collection data in the field (data is not shown), to reflect the natural density of grasshopper nymphs at our field site (about two-three nymphs per three individual plants). Once the experiment was concluded, the grasshoppers were removed. Same plant measurements, as described for the field experiment, were taken at two time points, at the beginning and 3 weeks later, at the end of the herbivore assay.

### 4.4. Statistical Analysis

Each measurement of plant tolerance (i.e., plant biomass, plant height, and the number of leaves), for each *M. sinensis* cultivar, was compared between time points (in the field experiment) and between the beginning and the end of the herbivory assay (in the cage experiment) using separate one-way repeated measures ANOVA, followed by post hoc TukeyHSD test. In each test (i.e., for each *M. sinensis* cultivar), each plant tolerance measurement, for each plant individual, was treated as a “within-subjects” variable and was separated by time; the “subject/time” ratio was used as the error function. Due to lack of normality, the leaf damage data was compared using the Kruskal–Wallis test followed by post hoc Dunn test (the normality and heterogeneity of data were determined using the Shapiro–Wilk test and Bartlett’s test, respectively, at a = 0.05). For each measurement, in both the field and cage experiments, the significance level was adjusted using Benjamini–Hochberg critical values (see the footnote in Table 1). All analyses were conducted in R (v.4.1.0), and package dunn.test was used for pairwise comparisons using Dunn test [43].

## Figures and Tables

**Table 1:** Changes in plant responses to herbivory in five *Miscanthus sinensis* cultivars during the season June–August 2018 in the field and cage experiments.

Experiment	Parameter	*Miscanthus sinensis* Cultivars	One-Way Repeated Measures ANOVA/ Kruskal–Wallis Rank Sum Test		(*i*/*m*) × Q
Field experiment	Plant biomass as [height × number of leaves]	“Autumn Anthem”	*F*_3,110_ = 20.83	*p* < 0.001 *	0.008
		“Dixieland”	*F*_3,84_ = 8.64	*p* < 0.001 *	0.008
		“Gracillimus”	*F*_3,99_ = 28.77	*p* < 0.001 *	0.008
		“Morning light”	*F*_3,114_ = 10.4	*p* < 0.001 *	0.008
		“Zebrinus”	*F*_3,116_ = 1.3	*p* = 0.278	0.025
	Height (cm)	“Autumn Anthem”	*F*_3,111_ = 3.35	*p* = 0.021 *	0.03
		“Dixieland”	*F*_3,85_ = 1.38	*p* = 0.255	0.05
		“Gracillimus”	*F*_3,99_ = 6.56	*p* < 0.001 *	0.008
		“Morning light”	*F*_3,114_ = 1.93	*p* = 0.128	0.04
		“Zebrinus”	*F*_3,116_ = 4.72	*p* = 0.004 *	0.025
	Number of leaves	“Autumn Anthem”	*F*_3,111_ = 25.33	*p* < 0.001 *	0.008
		“Dixieland”	*F*_3,84_ = 15.87	*p* < 0.001 *	0.008
		“Gracillimus”	*F*_3,99_ = 41.56	*p* < 0.001 *	0.008
		“Morning light”	*F*_3,114_ = 9.42	*p* < 0.001 *	0.008
		“Zebrinus”	*F*_3,116_ = 2.05	*p* = 0.11	0.025
	Leaf damage (%)	“Autumn Anthem”	χ^2^ = 53.99, df = 2	*p* < 0.001 *	0.008
		“Dixieland”	χ^2^ = 19.77, df = 2	*p* < 0.001 *	0.008
		“Gracillimus”	χ^2^ = 27.04, df = 2	*p* < 0.001 *	0.008
		“Morning light”	χ^2^ = 41.99, df = 2	*p* < 0.001 *	0.008
		“Zebrinus”	χ^2^ = 12.27, df = 2	*p* = 0.002 *	0.025
Cage experiment	Plant biomass as [height × number of leaves]	“Autumn Anthem”	*F*_1,28_ = 5.1	*p* = 0.03 *	0.04
		“Dixieland”	*F*_1,28_ = 0.73	*p* = 0.39	0.05
		“Gracillimus”	*F*_1,28_ = 9.06	*p* = 0.005 *	0.008
		“Morning light”	*F*_1,28_ = 7.61	*p* = 0.01 *	0.03
		“Zebrinus”	*F*_1,28_ = 10.55	*p* = 0.003 *	0.025
	Height (cm)	“Autumn Anthem”	*F*_1,28_ = 0.12	*p* = 0.72	0.04
		“Dixieland”	*F*_1,28_ = 0.38	*p* = 0.54	0.025
		“Gracillimus”	*F*_1,28_ = 0.15	*p* = 0.69	0.03
		“Morning light”	*F*_1,28_ = 0.05	*p* = 0.82	0.05
		“Zebrinus”	*F*_1,28_ = 0.62	*p* = 0.43	0.008
	Number of leaves	“Autumn Anthem”	*F*_1,28_ = 3.94	*p* = 0.06	0.03
		“Dixieland”	*F*_1,28_ = 0.69	*p* = 0.41	0.04
		“Gracillimus”	*F*_1,28_ = 11.24	*p* = 0.002 *	0.008
		“Morning light”	*F*_1,28_ = 11.49	*p* = 0.002 *	0.008
		“Zebrinus”	*F*_1,28_ = 7.27	*p* = 0.01 *	0.025
	Leaf damage (%)	“Autumn Anthem”	χ^2^ = 35.56, df = 4	*p* < 0.001 *	
		“Dixieland”			
		“Gracillimus”			
		“Morning light”			
		“Zebrinus”			

*p* values with asterisks (‘‘*’’) are significant at the corresponding Benjamini–Hochberg critical values [(*i*/*m*) × Q; where Q = 0.05, *i* is a *p*-value rank, and *m* is the total number of different parameter types used].

## Data Availability

All the raw data collected during the study are available upon request.
